# Impact of Bilateral Coordinated Movement on Manipulative Skill Competency in Elementary School Students

**DOI:** 10.3390/children8060517

**Published:** 2021-06-18

**Authors:** Jun Chen, Xiaozan Wang, Weiyun Chen

**Affiliations:** 1 College of Physical Education and Health, East China Normal University, Shanghai 200241, China; michaelsara2011@163.com (J.C.); xzwang@tyxx.ecnu.edu.cn (X.W.); 2 Research on Physical Education for Adolescents of Shanghai Social Science Innovation Research Base, Shanghai 200241, China; 3 Key Laboratory of Adolescent Health Assessment and Exercise Intervention of Ministry of Education, East China Normal University, Shanghai 200241, China; 4 School of Kinesiology, University of Michigan, 830 N University Avenue, Ann Arbor, MI 48109, USA

**Keywords:** soccer skills competency, basketball skills competency, midline crossing movements, skill assessment

## Abstract

Background: Researchers have found that manipulative skill competency in childhood not only helps to improve physical activity participation but also helps adolescents learn specialized sports skills. This study aimed to examine the effects of an eight-week bilateral coordinated movement (BCM) intervention on manipulative skill competency in school-aged children. Methods: The participants were 314 fourth-grade students from two elementary schools in China. This study used a two-arm quasi-experimental research design. For one elementary school, two fourth-grade classes were assigned to the BCM group and another two fourth-grade classes were assigned to the control group. For the other elementary school, one fourth-grade class was assigned to the BCM group and another fourth-grade class to the control group. The students in the BCM group received an eight-week, two 40 min BCM lessons in soccer, and another eight-week, two 40-min BCM lessons in basketball. The control group received an eight-week two regular 40 min PE lessons in soccer and basketball, respectively. The students’ manipulative skill competency in soccer and basketball skills were pre- and post-tested using the two PE metric assessment rubrics. Data were analyzed by means of descriptive statistics, independent sample t-tests, and ANCOVA and ANOVA repeated measures. Results: The results showed a significant main effect of time (pre-test vs. post-test) in soccer skills (*F* = 273.095, *p* < 0.001, *η*^2^ = 0.468) and in basketball skills (*F* = 74.619, *p* < 0.001, *η*^2^ = 0.193). Additionally, the results revealed a significant main effect of the group (BCM group vs. control group) in soccer skills (F = 37.532, *p* < 0.001, *η*^2^ = 0.108) and a marginal significant main effect of the groups in basketball skills (*F* = 3.619, *p* = 0.058, *η*^2^ = 0.011). Furthermore, there was a significant interaction effect between the time and the group in soccer skills (*F* = 37.532, *p* < 0.001, *η*^2^ = 0.108) and in basketball skills (*F* = 18.380, *p* < 0.001, *η*^2^ = 0.056). Conclusions: It was concluded that after participation in the eight-week, 16 40 min lessons of BCM, the fourth-grade students showed greater improvement in soccer and basketball dribbling, passing and receiving skills, compared to the control group.

## 1. Introduction

Studies have proven that motor skill competency, such as locomotor skills and manipulative skills, have a positive relationship with physical activity in childhood and adolescence [1]. Manipulative skill competency is referred to as the ability to perform skills that use the hands, feet, other body parts, and objects to manipulate or control an object (e.g., dribbling a ball with the hands, throwing a ball overhand, striking a ball with a racket) with proper forms consistently and proficiently [2,3,4,5]. Developing manipulative skill competency in childhood is instrumental for school-aged children to be physically active, especially for increasing participation in physical activity in later life [5,6,7,8,9]. Barnett et al. [9] found that Australian children’s motor skill proficiency, especially manipulative skill proficiency, predicted adolescent physical activity. Thus, Barnett et al. [9] suggested that in the development of elementary school students’ motor skill competency, more attention should be paid to improving manipulative skills than locomotor skills. Given the fact that the majority of youth are insufficiently active [10,11,12,13], it is important to improve manipulative skill competency in childhood to increase participation in physical activity.

The goal of quality physical education (PE) is to develop physically educated individuals who have the knowledge, skills, and confidence to enjoy a lifetime of physical activity [14]. In line with this, helping students to develop manipulative skill competency is one of the desired learning outcomes based on the National Physical Education Content Standard 1 [14]. Studies have shown that quality PE programs can improve manipulative skill competency in school-aged children [15,16,17]. McKenzie et al. [15] found that quality PE programs, delivered by certified PE teachers and classroom teachers with extensive training, can effectively improve American children’s manipulative skills. Chen et al. [16] found that the overall quality of PE teaching, and the four essential dimensions of quality PE teaching practices (i.e., task design, task presentation, class management, and instruction guidance), significantly contributed to developing American students’ manipulative skill competency. Bellows et al. [17] found that Mighty Moves (a fundamental motor skill intervention program) implemented by classroom teachers can improve at-risk American preschool children’s manipulative skills. In conclusion, the development of children’s manipulative skill competency is based on the interaction of the environment and the tasks [18].

Empirical studies have shown that bilateral coordinated movements contributed to improved manipulative skill competency [19,20,21]. Coordinated bilateral activities refer to the use of both the hands or the feet, and other parts of the body, to perform movements that cross the midline of the body [22,23]. Manual midline crossing movements involve crossing the midline of the body and manipulating objects in the opposite space so that both hands are used [24,25]. Basketball dribbling is a typical skill crossing the body’s midline. For example, crossover dribbling is performed from right to left, left to right. Cermak, et al. [26] investigated the ability of 150 American children, aged four to eight years old, to cross the body midline. The results found that the Space Visualization Contralateral Use (SVCU) scores of the children increased with age [26]. Incorporating midline crossing movements into sports may help children achieve greater motor skill performance [27]. For example, when a basketball player dribbles while changing directions to beat the defense, he or she must be able to use both hands to dribble the ball. Similarly, when a soccer player breaks through a defender, he or she must be able to dribble with both dominant and non-dominant feet. In addition, the previous studies found that bilateral coordinated movement practices were beneficial for children to improve dribbling and kicking skills in soccer performance [19], dribbling and shooting skills in basketball [20,21], and throwing skills [25]. Some studies have focused on examining the effects of the bilateral coordinated movement (BCM) on improving children’s manipulative skill competency.

To the best of our knowledge, there is a lack of study examining the effects of BCM PE lessons on fourth-grade students’ manipulative skill competency in China. Therefore, the purpose of this study is to examine the effects of the BCM intervention on manipulative skill competency in fourth-grade students who were assessed with the selected PE metrics assessment rubrics [28]. Our research hypothesis is that students in the BCM intervention group would show a significantly greater increase in their manipulative skill competency when assessed with the PE metric assessment rubrics when compared to the control group. The significance of this study lies in providing empirical evidence for the BCM intervention in improving students’ manipulative skill competency during PE lessons.

## 2. Methods

### 2.1. Participants and Research Settings

The participants in this study were 314 fourth-grade Chinese students aged 9–11 years old (boys = 182; girls = 132; mean age = 9.75 years old; standard deviation = 0.62) from two elementary schools during the school year of 2018–2019. Based on our previous collaboration, we recruited the two schools to participate by sending the overview of the study, including the study purpose, teacher training, intervention components, outcome measures, and timelines for intervention and outcome assessment, to PE teachers via the widely used social media in China, followed by addressing questions and concerns. Once the PE teachers agreed to participate, we sent a formal invitation letter including all of the key aspects of the study procedures to each school principal. We received the two approval letters indicating.

A two-arm quasi-experimental design was used in this study. For one elementary school, we assigned two fourth-grade classes to the BCM group, and another two fourth-grade classes to the control group. For the other elementary school, we allocated one fourth-grade class to the BCM group, and another fourth-grade class to the control group. At both of the two elementary schools, each participating fourth-grade class, regardless of the conditions, engaged in three 40 min lessons of PE per week. The PE class size ranged from 48–55 students. The PE teachers’ ages varied from 27–47 years old, with a range of 7–30 years of teaching experience. Each school’s principal granted permission for their school to participate in this study. The university’s Institutional Review Board (HUM00149529) approved the study protocols. Each student’s parent/guardian signed the consent form to allow their children to participate in this study. All four PE teachers signed the consent form to indicate their voluntary participation in this study.

### 2.2. Treatment

BCM group. The students in the BCM group received 16 40 min BCM lessons in soccer and 16 40 min BCM lessons in basketball during the fall semester of 2018 and the spring semester of 2019. To ensure the success of each intervention, the PE teacher implemented the key intervention content in each BCM lesson that we designed. We prepared 16 structured BCM soccer lesson plans as an eight-week unit and 16 BCM basketball lesson plans as an eight-week unit based on the school’s scheduled length of each PE lesson. Each structured BCM lesson plan consisted of three key content components: (1) 5–8 mins of bilateral coordinated aerobic movements as warm-ups; (2) 15–20 mins of sequential skill-building tasks focusing on bilateral coordinated motor skills including manipulative skills (i.e., dribbling, catching, passing, shooting) in combination with locomotor skills (i.e., running, jumping, skipping). The manipulative skills, along with the locomotor skills, used in playing sports and aerobic activities are core PE content aligned with the national PE content standards for grades 3–5 [14]. The students used bilateral, hand-eye, and multi-limb coordination to engage in the sequential skill-building tasks; and (3) 8–10 mins of skill-applying coordinated aerobic sports games tasks in which students applied skills in cognitively challenging, dynamic, and changing environments (e.g., play a dribble tag game with groups of four within a designated playing area). At the end of each lesson, there were 3–5 mins of lesson debriefing in which students self-reflected on their skill and game performance. While the intervention PE teachers were required to teach the same curriculum content within the same lesson structure, they had the flexibility to modify and adjust some of the specific learning tasks, the organization of them, as well as the pace of the task progression based on the class size, available facilities and equipment, and the students’ learning responses.

During the second to last week of July 2018, the corresponding investigator who designed the 16 BCM soccer lesson plans and the 16 BCM basketball lessons, trained the PE teachers in learning and teaching the sample BCM lessons in soccer and basketball during a 5-day BCM training course (6 h per day). Details in training are described elsewhere. During the fall semester, each trained PE teacher used the 16 BCM soccer lesson plans to teach 16 BCM soccer lessons (two 40 min lessons per week) to their students in the intervention classes for eight consecutive weeks. Similarly, during the spring semester, each trained PE teacher used the 16 BCM basketball lesson plans to teach 16 BCM basketball lessons (two 40 min lessons per week) to their students in the intervention classes for eight consecutive weeks.

Control group. The students in the control group followed their normal school curriculum schedules. During the fall semester of 2018, each control PE teacher used their lesson plans and usual teaching methods to teach 16 soccer lessons (two 40 min soccer lessons per week) to their students in the control classes for eight consecutive weeks. During the spring semester of 2019, each control PE teacher used their lesson plans and usual teaching methods to teach 16 basketball lessons (two 40 min basketball lessons per week) to their students in the control classes for eight consecutive weeks.

### 2.3. Motor Skill Assessments

The motor skill assessment for soccer was carried out by assessing the students’ performance levels according to the three essential dimensions: dribbling, passing and receiving, using a 0–4 rating scale. One trial was allowed for the test. Criteria for Competence (level 3) for dribbling is: “dribble with control while moving at a slow, consistent jog”. For passing it is: “sends a receivable lead pass to a partner so it can be received outside the passing lane without a break in the receiver’s stride on at least three passes,” and for receiving it is: “moves forward and outside the passing lane to meet the ball while receiving at least three receivable passes” [28] (p. 120). A total score of 9 indicated an Overall Competent Level. The maximum score is 12.

The motor skill assessment for basketball was carried out by assessing the students’ performance levels according to the three essential dimensions: dribbling, passing and receiving, using a 0–4 rating scale. One trial was allowed for the test. Criteria for Competence (level 3) for dribbling is: “dribbles with control while moving at a slow, consistent jog.” For passing it is: “sends a catchable lead pass to a partner so it can be caught outside the passing lane without a break in the receiver’s stride on at least three passes,” and for receiving it is: “moves forward and outside the passing lane to meet the ball while receiving at least three receivable passes” [28] (p. 98). A total score of 9 indicated an Overall Competent Level. The maximum score is 12.

Prior to the beginning of the fall semester, both intervention and control PE teachers were trained in learning the two PE metrics assessment rubrics, assessment criteria, assessment tasks, the testing protocols for soccer and basketball skills, and how to organize the students to perform the soccer skill and the basketball skill assessment tasks. During the fall semester, for the pre-test, the PE teachers organized the participating students to perform soccer dribbling, passing, and receiving skills in regular PE lessons during the last two weeks of September. For the post-test, the PE teachers organized the students to perform the same skills in regular PE lessons during the second and third weeks of December. During the spring semester, the students were pre-tested in basketball dribbling, passing, and receiving skills during the first two weeks of March, which was organized by the PE teachers. Again, the students were post-tested on the same basketball skills as above during the last two weeks of May, which was organized by the PE teachers. During the testing lessons, first, the PE teacher demonstrated and explained how to carry out the test based on the PE metric testing directions. Next, the PE teacher organized the students into pairs and gave each pair two practicing trials to ensure all of the students understood the testing procedures. Lastly, the PE teacher organized each pair to take the test, which was video-recorded using a digital camera.

In this study, the Cronbach’s alpha reliability coefficients of the soccer skills and the basketball skills assessments were 0.90, 0.82, respectively. The results showed that the two manipulative skill assessments had satisfactory internal consistency reliability [29].

### 2.4. Evaluation of Students’ Performance in Soccer and Basketball Skills

Prior to evaluating the students’ performance, in both the soccer and the basketball skills, four evaluators were trained in the use of the PE metrics assessment rubrics to assess each student’s performance of the soccer and the basketball skills by the corresponding author. Then, the four evaluators, who were paired up, began to practice coding six 4th-grade students’ performances in the soccer and the basketball skills, respectively, which were video-recorded not for this study. Each pair practiced coding for approximately three hours while discussing questions about the coding protocols spontaneously and finalized coding protocols. Next, each student in each pair independently coded the four students’ performance in the soccer and the basketball skills with the PE metrics assessment sheet to check the inter-rater reliability (IR). The IR of the coded soccer or basketball skills assessments was examined by checking each evaluators coding results using the formula: % IR = (number of agreement ÷ (numbers of agreement + numbers of disagreement)) × 100 [30]. Finally, when the IR of the three coded soccer or basketball skills was above 80%, the four evaluators began to officially code all of the video-recorded students’ performances in both the soccer and the basketball skills with the PE metric assessment sheet using the coding protocols. The two evaluators in each pair watched the recorded video together, but each evaluator independently coded each video-recorded student performance in both the soccer and the basketball skills. Each evaluator was strictly required to use the PE metrics assessment rubrics [28] to conduct the two manipulative skill assessments with all of the participating students.

### 2.5. Data Analysis

SPSS statistics software (Version 26.0; IBM Cooperation, Armonk, NY, USA) was used to conduct all statistical analyses of the data. To determine the levels and the proportions of the boys’ and the girls’ demonstrations of competency in two manipulative skill assessments, descriptive statistics and percentages were computed. To examine the pre-test mean score differences in each skill assessment, between the BCM and the control groups, the independent sample *t* test was conducted. The independent t-test showed, in the pre-test, a significant difference in the mean score of the Overall Competent Level in the soccer skills between the two groups (*t* = −7.013, *p* < 0.001). To examine the intervention effects on overall soccer skill competency, a 2 × 2 mixed-design analysis of covariance (ANCOVA) with repeated measures was conducted. In the ANCOVA repeated measure analysis, the time (pre-test vs. post-test) was treated as a within-subject factor, and the treatment condition (BCM group vs. control group) was treated as a between-subject factor. The pre-test score in soccer was a covariate to control for baseline differences.

In the pre-test, due to no significant difference in the mean score of the Overall Competent Level in basketball skills between the two groups (*t* = −1.021, *p* = 0.308), the ANOVA repeated measure was conducted with the time (pre-test vs. post-test) as a within-subject factor and the group (BCM group vs. control group) as a between-subject factor to examine if there was a significant intervention effect on basketball skill competency between the two groups. An alpha level of 0.05 was set for all statistical analyses.

## 3. Results

### 3.1. Soccer Skill Assessments

Table 1 presents the descriptive statistics of the pre-test and the post-test in the soccer skill assessment between the two groups and by gender. For the soccer dribbling, passing, and receiving skills assessment, a total score of 9 indicated the Overall Competent Level. As seen in Table 1, in the pre-test, 23% of 90 boys and 25% of 69 girls in the BCM group, demonstrated the Overall Competent Level or above in soccer skills. In contrast, 68% of 92 boys and 55% of 62 girls in the control group, demonstrated the Overall Competent Level or above in soccer skills. In the post-test, 49% of boys and 62% of girls in the BCM group demonstrated the Overall Competent Level or above in soccer skills. Conversely, 51% of boys and 37% of the girls in the control group demonstrated the Overall Competent Level or above in soccer skills.

As presented in Table 2, the results of the ANCOVA repeated measure showed a significant main effect of time (*F* = 273.095, *p* < 0.001, *η*^2^ = 0.468), a significant main effect of the group (*F* = 37.532, *p* < 0.001, *η*^2^ = 0.108), and a significant interaction between the time and the group (*F* = 37.532, *p* < 0.001, *η*^2^ = 0.108) in the overall soccer skill competency score. The results indicated that when the pre-test scores of the two groups were controlled, the BCM group showed a significant improvement in the overall soccer skill competency from the pre-test to the post-test, while the control group reduced the overall soccer skill competency over time.

### 3.2. Basketball Skill Assessments

Table 1 presents the descriptive statistics of the pre- and post-test in basketball skill assessment between the two groups and by gender. For the basketball dribbling, passing and receiving skill assessment, a total score of 9 indicated the Overall Competent Level. At the pre-test, 6% of 89 boys and 3% of 69 girls in the BCM group demonstrated the Overall Competent Level or above in the basketball skills. Similarly, 4% of 93 boys and 3% of 63 girls in the control group demonstrated the Overall Competent Level or above in the basketball skills. However, in the post-test, 46% of boys and 17% of girls in the BCM group demonstrated the Overall Competent Level or above in the basketball skills, while 14% of boys and 10% of girls in the control group demonstrated the Overall Competent Level or above in the basketball skills.

As presented in Table 2, the results of the ANOVA repeated measure revealed a significant main effect of time (*F* = 74.619, *p* < 0.001, *η*^2^ = 0.193), a marginal significant effect of the group (*F* = 3.619, *p* = 0.058, *η*^2^ = 0.011), and a significant interaction between time and the group (*F* = 18.380, *p* < 0.001, *η*^2^ = 0.056). The BCM group showed a significant increase in the basketball skill competency from the pre-test to post-test, compared to the control group. The results revealed a significant intervention effect in improving the basketball skills competency.

## 4. Discussion

This study is central to examining the effects of the BCM interventions on manipulative skill competency in the soccer and the basketball skills among children aged 9–11 years old. After the eight-week intervention, the students in the BCM group demonstrated a significant improvement in the two manipulative skills over time, compared to their counterparts in the control group. In addition, in the BCM group, 55% of students demonstrated an Overall Competent Level or above in the soccer skills and 34% of students demonstrated an Overall Competent Level or above in the basketball skills in the post-test. Our results confirm that BCM in PE lessons can improve students’ manipulative skill competency.

Consistent with the results in the present study, previous studies have shown that bilateral coordinated practice improves manipulative skills in children and adolescents [19,20,21]. For example, Teixeira et al. [19] found that 24 Brazilian adolescents aged 12–14 years old, participating in five, two-hour bilateral practice lessons per week over the course of four months, helped to reduce the lateral asymmetries of performance assessed on three soccer skills: kicking for force, kicking for accuracy, and the speed of dribbling. Teixeira et al. [19] also suggest that spending more time on non-dominant limbs is conducive to achieving good motor skill performance. Further, Stöckel et al. [20] found that 52 sixth- and seventh-grade German students, who participated in eight 45 min bilateral practices of hand dribbling skills over four weeks, showed significant improvement in basketball dribbling skills, compared to the comparison students. In addition, Pedersen [27] found that just 30 mins of specific midline crossing coordinated activities such as two-handed ball bouncing or catching games improved Australian children’s processing speed of lateral movements. However, this study has not focused on learning actual sports skills.

The finding of this study indicates that students, who participated in the BCM throughout the 16 lessons, significantly improved their overall skill competency in both the soccer and the basketball skills. In order to improve the students’ manipulative skills, we designed soccer and basketball lesson plans including three key components: bilateral coordinated aerobic movements (warm-ups), skill-building tasks focusing on bilateral coordinated manipulative skills in combination with locomotor skills, and skill-applying coordinated aerobic sports games tasks. The organismic interactions with the task and the environment plays an important role in the development of motor skills [18,31]. Additionally, Logan et al. [32] indicated that motor skill interventions are effective strategies to improve children’s motor skill competency.

Dribbling, passing, and receiving skills in soccer and basketball are basic specialized sports skills often used in playing soccer and basketball games. Most importantly, mastering these basic specialized skills during childhood is beneficial to their later motor skills learning. The fundamental movement skills are associated with organized physical activity in adolescents [7,33]. Due to the trend of decreasing physical activity participation in youth [10,11,12,13], it is important to improve children’s manipulative skill competency. This study suggests that to better equip children with manipulative skill competency in sports-related skills, PE teachers should devote their instructions to the bilateral coordinated movement practice in basic specialized skills. For example, in this study, in order to improve basketball dribbling skills, we designed a variety of dribbling activities (e.g., dribbling while walking, crossover dribbling while walking, dribbling while running, dribbling on zigzag pathways, and dribbling at different speeds, etc.) combined with bilateral coordinated practices such as dribbling while walking or running on a straight pathway with different hands, or dribbling while walking or running on curved pathways while changing direction and hands.

It is important to note several implications associated with the findings of this study. First, this is the first study that has attempted to examine the effects of BCM on manipulative skill competency in elementary school students who were assessed with selected PE metric assessment rubrics. The results of this study show that students significantly improved their manipulative skill competency through participating in the structured BCM lessons. Second, this study adds to the literature on manipulative skill assessment with the PE metrics assessment rubrics. This study suggests that the PE metrics assessments are useful tools for measuring students’ manipulative skill competency in future studies. Third, given the promising results of BCM in this study, future studies may consider designing BCM intervention studies to improve other manipulative skill competencies such as striking skills, throwing skills, passing skills, and catching skills among school-aged children.

The limitation of the study was that a quasi-experimental design was used. The BCM group and the control group were allocated based on the choice of the PE teachers. Given that, the study could not control for the baseline differences in the Overall Competency Score in the soccer skills between the two groups, with the control group having a significantly higher score. In addition, this study was limited to the use of BCM as the intervention strategy to improve manipulative skill competency in the soccer and the basketball skills. Future studies could examine BCM on other manipulative skill competencies (e.g., overhand throwing, striking with a paddle) and locomotor skill competencies (e.g., jumping, dance) in school-aged children.

## 5. Conclusions

Our study found that the students in the BCM group showed a significant improvement in manipulative skill competency assessed with the PE metrics assessment rubrics when compared to the control group. This study suggests that engaging students in BCM is an effective strategy to improve soccer and basketball skills in children aged 9–11 years old. Future research may examine the effects of the BCM intervention on other manipulative skill competencies such as throwing or striking in children who will be assessed with the PE metrics assessment rubrics.

## Figures and Tables

**Table 1:** Manipulative skill assessment performance of the students in the two cohorts.

	BCM		Control	
	M (SD)	# of OCL (% of OCL)	M (SD)	# of OCL (% of OCL)
Soccer (competence score: 9)				
Pre-test				
Boys (*n* = 90)	5.78 (3.90)	21 (23%)	9.14 (2.50)	63 (68%)
Girls (*n* = 69)	6.07 (3.42)	19 (25%)	7.65 (3.26)	34 (55%)
Total (*n* = 159)	5.91 (3.69)	40 (25%)	8.54 (2.92)	97 (63%)
Post-test				
Boys (*n* = 92)	8.13 (3.14)	44 (49%)	8.30 (2.52)	46 (51%)
Girls (*n* = 62)	8.65 (2.40)	43 (62%)	7.60 (2.27)	23 (37%)
Total (*n* = 154)	8.36 (2.84)	87 (55%)	8.02 (2.43)	69 (45%)
Basketball (competence score: 9)				
Pre-test				
Boys (*n* = 89)	5.65 (2.17)	5 (6%)	5.39 (2.33)	4 (4%)
Girls (*n* = 69)	3.75 (2.44)	2 (3%)	4.67 (2.11)	2 (3%)
Total (*n* = 158)	4.82 (2.47)	7 (4%)	5.10 (2.26)	6 (4%)
Post-test				
Boys (*n* = 93)	7.96 (2.86)	41 (46%)	6.23 (2.17)	13 (14%)
Girls (*n* = 63)	5.80 (3.14)	12 (17%)	5.25 (2.50)	6 (10%)
Total (*n* = 156)	7.01 (3.16)	53 (34%)	5.83 (2.35)	19 (12%)

Note: BCM = bilateral coordinated movement; OCL = Overall Competent Level; M = Mean, SD = Standard deviation; # = Number sign.

**Table 2:** Results of the repeated measure of the ANCOVA and the ANOVA for manipulative skills between the two cohorts.

Dependent Variables	Factors	F	df	P	*η* ^2^	
Soccer	Time	273.095	1	<0.001	0.468	
	group	37.532	1	<0.001	0.108	
	Time*Group	37.532	1	<0.001	0.108	
Basketball	Time	74.619	1	<0.001	0.193	
	group	3.619	1	0.058	0.011	
	Time*Group	18.380	1	<0.001	0.056	

Note: F = the F-value; df = degrees of freedom; P = statistical significance; *η*^2^ = Eta squared.

## Data Availability

The data presented in this study are available on request from the corresponding author.
